# Dietary Inflammatory Index and Non-Communicable Disease Risk: A Narrative Review

**DOI:** 10.3390/nu11081873

**Published:** 2019-08-12

**Authors:** Catherine M. Phillips, Ling-Wei Chen, Barbara Heude, Jonathan Y. Bernard, Nicholas C. Harvey, Liesbeth Duijts, Sara M. Mensink-Bout, Kinga Polanska, Giulia Mancano, Matthew Suderman, Nitin Shivappa, James R. Hébert

**Affiliations:** 1 HRB Centre for Diet and Health Research, School of Public Health, Physiotherapy, and Sports Science, University College Dublin, Belfield, Dublin 4, Ireland; 2 HRB Centre for Diet and Health Research, School of Public Health, University College Cork, Western Gateway Building, Western Rd, Cork, Co. Cork, Ireland; 3 Research Team on the Early Life Origins of Health (EAROH), Centre for Research in Epidemiology and Statistics (CRESS), INSERM, Université de Paris, F-94807 Villejuif, France; 4 MRC Lifecourse Epidemiology Unit, University of Southampton, Southampton General Hospital, Southampton SO16 6YD, UK; 5 The Generation R Study Group, Erasmus MC, University Medical Center, P.O. Box 2040, 3000 CA Rotterdam, The Netherlands; 6 Department of Pediatrics, Division of Respiratory Medicine and Allergology, Erasmus MC, University Medical Center, P.O. Box 2060, 3000 CB Rotterdam, The Netherlands; 7 Department of Pediatrics, Division of Neonatology, Erasmus MC, University Medical Center, P.O. Box 2060, 3000 CB Rotterdam, The Netherlands; 8 Department of Environmental Epidemiology, Nofer Institute of Occupational Medicine, 91-348 Lodz, Poland; 9 MRC Integrative Epidemiology Unit, Population Health Sciences, Bristol Medical School, University of Bristol, Bristol BS8 2BN, UK; 10 Cancer Prevention and Control Program and Department of Epidemiology and Biostatistics, Arnold School of Public Health, University of South Carolina, Columbia, SC 29208, USA; 11 Connecting Health Innovations LLC, Columbia, SC 29201, USA

**Keywords:** dietary inflammatory index, inflammation, cardiometabolic health, obesity, metabolic syndrome, cancer, respiratory health, bone health, mental health, neurodevelopment

## Abstract

There are over 1,000,000 publications on diet and health and over 480,000 references on inflammation in the National Library of Medicine database. In addition, there have now been over 30,000 peer-reviewed articles published on the relationship between diet, inflammation, and health outcomes. Based on this voluminous literature, it is now recognized that low-grade, chronic systemic inflammation is associated with most non-communicable diseases (NCDs), including diabetes, obesity, cardiovascular disease, cancers, respiratory and musculoskeletal disorders, as well as impaired neurodevelopment and adverse mental health outcomes. Dietary components modulate inflammatory status. In recent years, the Dietary Inflammatory Index (DII^®^), a literature-derived dietary index, was developed to characterize the inflammatory potential of habitual diet. Subsequently, a large and rapidly growing body of research investigating associations between dietary inflammatory potential, determined by the DII, and risk of a wide range of NCDs has emerged. In this narrative review, we examine the current state of the science regarding relationships between the DII and cancer, cardiometabolic, respiratory and musculoskeletal diseases, neurodevelopment, and adverse mental health outcomes. We synthesize the findings from recent studies, discuss potential underlying mechanisms, and look to the future regarding novel applications of the adult and children’s DII (C-DII) scores and new avenues of investigation in this field of nutritional research.

## 1. Introduction

Non-communicable diseases (NCDs) are a major contributor to the global burden of disease and account for up to 72% of worldwide deaths [1]. Chronic low-grade inflammation, characterized by persistent elevated concentrations of circulating pro-inflammatory cytokines, across the life span has been associated with the development of both age and diet-related NCDs, including obesity, cardiometabolic diseases, many cancers, respiratory and auto-immune disorders, arthritis, and depression [2,3,4]. The link between dietary habits and NCDs has been extensively examined [5,6]. Many NCDs are, to a large extent, preventable and modifiable lifestyle-related risk factors including an unhealthy diet play a significant role [7,8,9,10,11,12]. The Global Burden of Diseases Nutrition and Chronic Diseases Expert Group examined global dietary quality trends among adults across 187 nations over 20 years (1990–2010). They reported a modest increase in the consumption of healthy foods over that time; however, this was surpassed by a greater increase in intake of unhealthy foods [13]. Furthermore this study highlighted the potential impact of suboptimal diet on NCD risk and mortality and the need to improve diet on a worldwide scale. 

It is now recognized that diet is an important modulator of chronic inflammation [14]. Current evidence on the influence of diet on inflammation is based on different research approaches; i.e., nutrient-based, food group-based or analyses of whole-diet patterns. Examination of dietary patterns and indices has become more popular given the ease with which these measures can be generated from existing dietary data. Additionally, considering that we eat complex combinations of foods, rather than individual nutrients and food groups, such approaches are also more translatable in terms of public health messaging. However some common and method-specific limitations exist [15]. The dietary inflammatory index (DII^®^) was developed to characterize the inflammatory potential of the diet. In contrast to dietary indices used in epidemiologic research which have traditionally been based on adherence to dietary guidelines or recommendations including the Dietary Approaches to Stop Hypertension [16], the Healthy Eating Index-2010, the Alternative Healthy Eating Index [17,18,19]; adherence to specific foods or gastronomy such as the Mediterranean Dietary Index [20,21,22]; or study-specific derivations from regression techniques such as principal components analysis or reduced rank regression [23,24,25], the DII design is based on a wider range of human populations, study designs, and nutritional assessment methods [26]. Furthermore, the DII also includes evidence from qualifying cell culture and animal experiments [27]. 

Since its development the DII has been widely studied in various disease contexts to test the hypothesis that dietary inflammation is a determinant of NCD risk and mortality. Consistent with previous reports, recent data from 2 large Spanish prospective studies (n = 18,566 participants in total) provide strong evidence that inflammatory mechanisms link unhealthy diet and premature death [7]. The authors additionally included their findings in a meta-analysis with 10 studies included in previous meta-analyses investigating DII and mortality. They reported a 23% increased risk of all-cause mortality comparing highest vs. lowest DII categories [7], confirming that a pro-inflammatory diet is linked to greater all-cause mortality risk. In this narrative review, we examine the recent literature regarding relationships between the DII and NCD risk. We distill key findings, discuss potential underlying mechanisms, and conclude with future directions and new avenues of investigation for this area of nutritional research. 

## 2. DII Development

**Figure 1:**
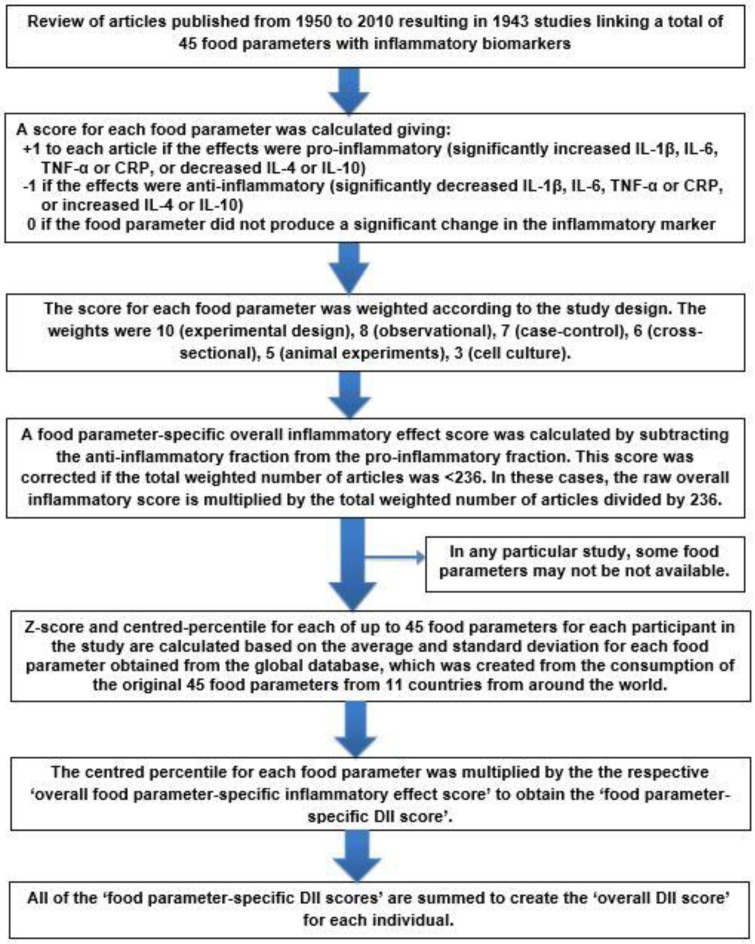
Sequence of steps in creating the Dietary Inflammatory Index (DII^®^).

Work to develop the DII began in 2004 and the paper describing the DII Generation (Gen) 1 method and construct validation was published in 2009 [26]. In 2014, we produced a refinement (Gen2) that included an improved scoring system, more complete literature search, and a comparative food and nutrient database that included data from 11 countries on four continents [27]. The entire process of developing the DII is described in our methods paper [27] and the steps involved are shown in Figure 1. The first step was to review all of the articles published through 2010 that studied at least one of the six inflammatory markers (i.e., c-reactive protein (CRP), interleukin (IL)-1β, IL-4, IL-6, IL-10, and tumor necrosis factor-α (TNF-α). Each food parameter was given a literature-derived inflammatory effect score based on scoring of articles, which depends on (1) study design, (2) direction of the association between the food parameter and inflammatory markers, and (3) strength and dose-response of the association. The final three steps involve relating an individual score to the global database comparison. This requires: (1) Z-scoring each parameter and then converting these Z-scores to a proportion and centering by doubling and subtracting 1; (2) multiplying each of these centered proportions by the respective overall food parameter-specific inflammatory effect score to obtain the food parameter-specific DII score; and (3) summing all of these food parameter-specific DII scores to create the overall DII score for each study participant.

Using methods of construct validation, we showed that the DII could predict interval changes in levels of high-sensitivity CRP (hs-CRP) in the Seasonal Variation in Blood Lipids (SEASONS) study in which dietary data were collected using both multiple (i.e., up to 15) 24 h recall interviews (24 h), five administrations of a structured questionnaire, the seven-day dietary recall [28], and up to five hs-CRP measures [29]. We have now tested the effect of diet-associated inflammation on inflammation markers, including CRP, IL-6, IL-4, and TNF-α receptor-2 [30,31,32,33,34,35,36]. Some of these studies were conducted with the sole purpose of validation [29,32,37]. In some studies examining DII and health outcome associations, validation was carried out as part of the larger study [38,39,40]. We also have tested effects on other markers of disease risk such as homocysteine, chemerin, fibrinogen, complement component C3, leukocytes/white blood cells, interferons, intercellular adhesion molecule, and vascular cell adhesion molecule 1 [31,33,41]. The DII or energy-adjusted DII (E-DII) have been construct validated against inflammatory biomarkers in different populations and under varying conditions [12,29,31,33,34,35,36,37,38,39,40,41,42,43,44,45,46,47,48]. More recently the children’s DII (C-DII) has been developed and construct validated with hs-CRP in an American study [49].

## 3. Methods

This is a narrative review of the current literature on the DII and NCD risk. The literature search performed in PubMed and Scopus included the following keywords, used separately and in combination: Dietary inflammatory index, NCD; cancer; type 2 diabetes, gestational diabetes, cardiovascular disease; cardiometabolic disease, obesity, metabolic syndrome, bone, musculoskeletal, neurodevelopment, mental health, depression, anxiety, respiratory health, and asthma. The inclusion criteria were peer-reviewed human studies, meta-analyses and systematic reviews from 2014 to May 31st 2019, so that the focus is on associations with the refined (i.e., generation 2) DII. Please note that all measures of association; e.g., odds ratios (ORs), hazard ratios (HRs), are adjusted for potential confounders, though these may vary in type and number across studies.

## 4. DII and Cancers

Since Virchow first proposed the connection between inflammation and cancer in the mid-19th century [50], interest in the relationship between factors that influence inflammation and cancer has continued to grow [51,52,53,54]. We now know that inflammation is not a single cancer-related mechanism. Rather, it is a substrate on which other mechanisms operate, including oxidative and nitrative DNA damage, changes in gene expression and genetic instability, insulin resistance, blunted immune response, neural signaling, and vascular dysfunction [55,56,57,58,59,60,61,62,63,64,65,66,67]. The DII concept arose in the context of conducting cancer research to address the conceptual and methodologic gaps between the burgeoning fields of inflammation-related and dietary research. Until the DII was developed there was no evidence-based tool to link diet-related inflammation to cancer or other health outcomes [15]. It is, therefore, no surprise that a major focus of DII research has been on its link with cancer. We have now published over 100 papers on the DII in relation to cancers of various anatomic/organ sites. In addition, other groups have published numerous papers based on the DII in relation to cancer, including several meta-analyses [51,52,53,54,68,69,70]. The vast majority of these publications have shown a positive association between increasing inflammatory potential of diet and cancer risk and mortality after a cancer diagnosis.

### 4.1. Colorectal Cancer (CRC)

Because of its strong association with inflammatory bowel disease (IBD), CRC has been known to be related to inflammation [71]. Furthermore, biomarkers of inflammation are known to be associated with CRC risk [72,73] and CRC’s connection with diet has been established for some time [74,75,76,77]. Therefore, it is not surprising that we have observed consistently strong positive relationships between the DII score and CRC. At this juncture, there are 13 papers published by our group on CRC incidence [42,78,79,80,81,82,83,84,85,86,87,88,89] from studies carried out in various populations across the world [81,82].

We have also published one meta-analysis [10], which showed that individuals in the highest versus the lowest (reference) DII category had an overall 40% increase in CRC risk of [relative risk (RR) = 1.40, 95% confidence interval (CI): 1.26, 1.55, *p* < 0.001]. Increase of this magnitude in a cancer that has the potential to kill has great public health significance. In the most recent publication from Spain [82], E-DII was associated with CRC risk in the highest, most pro-inflammatory E-DII quartile vs the lowest quartile (OR_E-DIIQuartile4vs1_ = 1.93, 95% CI: 1.60–2.32; P_trend_ < 0.001); this increase was observed for both colon and rectal cancer [82]. Because all risk estimates are based on exposures seen in actual populations, the measures of association actually underestimate potential risk because the variability we see within populations (usually around −5 to +5) is much less than the maximal theoretical range (around −9 to +8). When analyzed as a continuous variable, results showed a 7% increase in CRC risk for each 1-point increase in the DII score; this translates to more than doubling (119%) in risk for the most pro-inflammatory diet relative to the most anti-inflammatory. 

We have published three [90,91,92] papers on mortality in colorectal cancer survivors in relation to the DII or the E-DII. Results reflect a strong and consistently positive relationship between DII score and overall mortality (e.g., in the Women’s Health Initiative showed that women in the lowest tertile of E-DII scores from diet plus supplements has less than half the rate of all-cause mortality compared to those women in the highest, most pro-inflammatory, E-DII tertile (HR_Tertile1vs3_ = 0.49; 95% CI = 0.31–0.79) [92]. 

We also have published three papers on adenomatous polyps (CRC precursor lesions) and the DII. Results from these studies are not as consistently positive as those for CRC incidence, survival, or mortality. One of these, based on data from the Prostate, Lung, Colorectal, and Ovarian (PLCO) Cancer Screening Trial, showed a positive effect in both men and women, but with stronger associations in men (e.g., about a 40% increase in risk in quartile 4 (most pro-inflammatory diet) versus quartile 1 (OR_Quartile4vs1_ = 1.41; 95% CI = 1.23–1.62) [93]. Findings from another study in the US, focusing on recurrent colorectal adenomas, produced null effects [94]. The most recent study, from Iran, shows a consistent positive association, with greater than twice the odds of adenomas in the 3^rd^ vs 1^st^ tertile (OR_Tertile3vs1_ = 2.33; 95% CI: 1.30–4.02; P_trend_ = 0.005) [82]. Given the role of the microbiome in the colon and IBD [95] and the role of diet in modulating the microbiota [96,97], this is a frontier area that should be pursued. Recent evidence showing that the DII affects urinary enterolignans, compounds produced as a result of action by the microbiota, is very encouraging in this regard [39].

### 4.2. Cancers of the Upper Aerodigestive Tract

As a group, cancers of the upper aerodigestive tract, which include cancers of the larynx, nasopharynx, oral and pharynx, and esophagus, are strongly associated with tobacco, which, among its other properties, is known to be intensely pro-inflammatory [98,99]. Indeed, after CRC the most consistent, positive associations with DII scores have been for cancers of the upper aerodigestive tract. In general, these associations with DII are stronger than those observed for CRC. For example, three studies on laryngeal cancer [78,100,101] produced positive results, with more than a tripling of risk across DII quartiles in an Italian case-control study [100] (OR_Quartile4vs1_ = 3.30; 95% CI 2.06, 5.28; P_trend_ < 0.0001), and an even stronger effect in smokers (OR_Quartile4vs1_ = 4.86) and overweight subjects (OR_Quartile4vs1_ = 3.62). 

Studies on nasopharyngeal cancer [78,102,103] also produced positive results, with those in the highest tertile compared to subjects in the lowest DII tertile, having a >60% higher risk in Italy (OR _Tertile4vs1_ = 1.64; 95% CI: 1.06–2.55) [104]; while those in the highest DII quartile in Japan had a >4-fold higher risk relative to subjects with the most anti-inflammatory diets (OR_Quartile4vs1_ = 4.99; 95% CI: 1.14–21.79) [103]. Likewise, our one study on oral and pharyngeal cancer in Italy [104] showed that subjects with DII scores in the highest quartile were at nearly double the risk, of oral and pharyngeal cancer (OR_Quartile4vs1_ = 1.80; 95% CI 1.36–2.38). We also observed a strong combined effect of higher DII score and tobacco smoking or alcohol consumption on oral and pharyngeal cancer, which is consistent with the synergistic effect of alcohol, tobacco and a pro-inflammatory diet. 

Esophagus is the site where one would expect to see the strongest effect if there were actual mixing between dietary constituents and tobacco smoke condensates [105,106]. Indeed, we have seen the strongest associations between the DII and esophageal cancer [78,103,107,108,109,110,111]. For example, looking at esophageal squamous cell cancer in Iran, we observed a very strong linear effect of the DII (OR_continuous_ = 3.58, 95% CI: 1.76–7.26) [107]; in Italy a strong effect across quintiles (OR_quintile5vs1_ 2.46, 95 % CI 1.40–4.36; P_trend_ < 0.001) [108]; and an even stronger effect in Sweden (OR_Quartile4vs1_ 4.35, 95 % CI 2.24, 8.43) [109]. We also have observed strong associations with adenocarcinoma of the esophagus in Ireland (OR_Tertile3vs1_ = 2.29; 95% CI: 1.32–3.96) [110] and Sweden (OR_Quartile4vs1_ = 3.59, 95 % CI: 1.87–6.89) [109] and in overall esophageal cancer in China (OR_Quartile4vs1_ = 2.55; 95% CI: 1.61–4.06; P_trend_ < 0.001) [111].

### 4.3. Other Tobacco-Related Cancers

Given the ability of diet and tobacco to modulate inflammation, it would not be surprising to find strong associations between the DII and risk of other tobacco-related sites. Indeed, we have now observed associations between the DII/E-DII and lung cancer in the Melbourne Collaborative Cohort Study (n = 35,303) in Australia where the hazards ratio comparing quartile 4 to quartile 1 was (1.70 95% CI 1.02, 2.82, P_trend_ = 0.008] [112] and in current smokers (HR_Q5vsQ1_ = 1.44; 95% CI 1.11–1.86, P_trend_ = 0.03) and in male ever-smokers (HR_Quintile5vs1_ = 1.37; 95% CI 1.07–1.77; P_trend_ = 0.03) in Singapore [113]. However, in Sweden, the association was only marginally significant (with the reduction in HR_per-tertile decrease_ = 0.81; 95% CI 0.66–0.99) [114] and in Italy no relationship with the DII was observed, though there was radiological evidence of emphysema across DII quartiles (OR_Quartile4vs1_ = 1.30, 95% CI 1.01–1.67, P_trend_ = 0.01) [115]. 

We have also observed associations between the DII and urinary bladder cancer in analyses of data from elderly subjects in a pooled case-control in Italy (OR_continuous_ = 1.08, 95% CI: 1.00–1.17); P_trend_ = 0.003) [78] and in separate case-control studies in Italy (OR_Quartile4vs1_= 1.97, 95% CI: 1.28–3.03; P_trend_ = 0.003) [116] and Iran (OR_above vs below median_ = 2.46; 95% CI = 1.12–5.41) [117]. Two studies on renal cancer, both showing positive results, have been conducted in a prospective study of women in Iowa in the USA (HR_DIItertile3vs1_ = 1.52; 95% CI: 1.09, 2.13) [118] and in an Italian case-control study (OR_Quartile4vs1_ = 1.41, 95% CI: 1.02–1.97; P_trend_ = 0.04) [119].

### 4.4. Other Digestive Tract Sites 

We have also observed generally strong relationships between the DII and pancreatic cancer risk [78,120,121,122]. These associations are similar in scale to what we have observed for cancers of the aerodigestive tract. For example, extreme quintile comparisons from the patient registry supported by the Mayo Clinic Specialized Program of Research Excellence (SPORE) in Pancreatic Cancer at the Mayo Clinic showed extreme DII quintile comparisons of about 2.5-fold increased risk (OR_Quintile5vs1_ = 2.54, 95% CI: 1.87–3.46, P_trend_ < 0.0001) and even more extreme results among current smokers (OR_Quintile5vs1_ = 3.40, 95% CI: 2.28–5.07) and among participants with long-standing diabetes (OR_Quintile5vs1_ = 3.09, 95% CI: 2.02–4.72), highlighting the importance of underlying inflammatory conditions for cancer risk [121]. However, it should be noted that we also have observed null results in the PLCO Trial [123] and the NIH-AARP Diet and Health Study [124]. Other digestive tract sites for which we have results, albeit from relatively fewer studies, include the liver. One study from Italy showing results of about the same magnitude as other cancers in this group for hepatocellular cancer (OR_E-DII tertile 3vs1_ = 2.43, 95% CI: 1.27–4.68; P_trend_ = 0.03), with a stronger effect in hepatitis B- and C-negative participants (OR_E-DII tertile 3vs1_ = 4.18, 95% CI: 1.53–11.39; P_trend_ = 0.02) [125]. Similarly, in a Chinese case-control study we observed an effect about as large (OR_E-DIItertile3vs1_ = 3.22, 95% CI: 1.30–7.98, P_trend_ = 0.009) [126]. One study each from Italy (OR_Quartile4vs1_ = 2.35, 95% CI: 1.32–4.20; P_trend_ = 0.004) [127] and Iran (OR_above vs below median_ = 3.39; 95% CI = 1.59–7.22,) [38] showed positive associations between DII scores and gastric cancer.

### 4.5. Hormone-Sensitive Cancers

Of the hormone-sensitive cancers, prostate cancer has been among the most consistently positively associated with the DII, with 11 studies showing a positive relationship [78,128,129,130,131,132,133,134,135,136,137]. In a meta-analysis of the DII in relation to prostate cancer, the adjusted pooled RR of prostate cancer for the highest (the most pro-inflammatory diet) versus lowest (the most anti-inflammatory diet) DII categories was 1.74 (95% CI: 1.24–2.43) [68]. The analysis of the DII score as a continuous variable showed that the risk of prostate cancer was 9% higher for each one-point increase in the DII score [68]. We also have published one study on DII and survival from prostate cancer, which indicated that men with more poorly differentiated disease (Gleason score 7–10) were more like to die of all causes (HR_DIItertile3vs1_ = 2.88; 95% CI: 1.46–5.67) 2.88; 95% CI: 1.46–5.67) and prostate cancer (HR_DIItertile3vs1_ = 2.82; 95% CI: 1.17–6.80) [138]. Results from breast cancer incidence-focused studies tend to be consistent with a positive association between the DII and incident disease [139,140,141,142,143], but on the whole results have been modest compared to other cancers. For example, in the prospective Swedish Women’s Lifestyle Study there was about a 20% increase in being diagnosed with breast cancer across extreme DII quartiles (HR_DIIquartile4vs1_ = 1.18; 95% CI: 1.00–1.39), with somewhat stronger associations in postmenopausal women (HR_DIIquartile4vs1_=1.22; 95% CI: 1.01–1.46) [139]. In addition, we have observed null results in some studies [81,144,145]. Results from studies in the US and Italy focusing on breast cancer survival have been consistently positive [146,147,148]. Furthermore in the Women’s Health Initiative, E-DII scores were associated with a lower risk of CVD mortality (HR_Quartile1vsQ4_ = 0.44; 95% CI = 0.24–0.82; P_trend_ = 0.005), but not with breast cancer-specific mortality (HR_Quartile1vsQ4_ = 0.96; 95% CI = 0.62–1.49; P_trend_ = 0.96) or all-cause mortality (HR_Quartile1vsQ4_ = 0.82; 95% CI = 0.63–1.05; P_trend_ = 0.17) [146]. We have observed consistent positive results between the DII and ovarian cancer [149,150,151], including in African-American women, in whom we observed a 10% increase in epithelial ovarian cancer risk per one-unit change in the E-DII (OR = 1.10, 95% CI = 1.03–1.17) and in comparing women consuming the most pro-inflammatory diet compared to the most anti-inflammatory diet (OR_E-DIIQuartile4vs1_ =1.72; 95% CI = 1.18–2.51) [152]. In an Italian case-control study, women with the most pro-inflammatory diet had a higher risk for endometrial cancer compared with women with the most anti-inflammatory diet (OR_Quartile4vs1_ = 1.46, 95% CI: 1.02–2.11, P_trend_ = 0.04) [153]. 

### 4.6. Lymphomas

In two studies from Italy show higher that DII scores have been shown to be associated with an increased risk of Non-Hodgkin’s Lymphoma (OR_DIIQuartile4vs1_ = 1.61, 95% CI: 1.07–2.43; P_trend_ = 0.01) [154], but not with Hodgkin’s Lymphoma [155]. 

In summary, we have found consistent relationships between DII scores and cancer outcomes for a wide array of cancers. This is true across very diverse populations from around the world and using different dietary assessment methods—mostly different versions of food frequency questionnaires (FFQs). The most consistent results are for CRC, aerodigestive tract cancers, and prostate cancer. Studies that have produced null results tend to have shorter follow-up periods than most cohorts (e.g., the PLCO trial [123] and the NIH-AARP Diet and Health Study [124]) or case-control studies, which attempt to draw inferences between current diet and diet in the distant past.

## 5. DII and Cardiometabolic Health and Disease

Cardiometabolic disease encompasses a spectrum of conditions from insulin resistance progressing to clinically recognisable states of pre-diabetes, metabolic syndrome (MetS), and finally to cardiovascular disease (CVD) and type 2 diabetes mellitus (T2DM) [156]. These conditions are grouped under the same umbrella term as they share many risk factors such as dyslipidaemia, hypertension, and obesity [157]. Obesity is associated with low-grade inflammation within adipose tissue and the secretion of cytokine, leading to impaired lipid metabolism. It is worth noting that a substantial proportion of obese adults exhibit what has been described as a metabolically healthy obese phenotype, characterized by the absence of low-grade inflammation and other metabolic perturbations [158]. Other populations, including in East Asia (Korea) [159], South Asia (India) [160], West Asia (Iran) [161], and in African Americans [162], tend to be metabolically obese (i.e., they evince symptoms associated with obesity at lower levels of adiposity). CVD and T2DM are both leading causes of death [163] and pose substantial healthcare burdens worldwide. Chronic systemic inflammation, characterized by continuous elevated circulating levels of inflammatory cytokines, is implicated in the pathophysiology of CVD and T2DM [164,165,166,167,168]. As diet is a major determinant of inflammation, there is great interest in whether dietary inflammatory potential can modify an individual’s risk of cardiometabolic diseases. 

Recently, several (systematic) reviews and meta-analyses have been conducted to appraise evidence to date for the associations between DII and cardiometabolic disease [11,169,170,171]. Of these, three reviews focused on CVD, MetS, and mortality [11,170,171], while the other focused on obesity and body mass index (BMI) [169]. In general, a more pro-inflammatory diet, as indicated by a higher DII score, has been associated with higher risk of obesity [169], CVD risk, and CVD-related mortality [11,170,171], while the relationship between DII and MetS is less consistent [170,171].

### 5.1. DII and CVD Risk and CVD Mortality

Recent reviews unanimously concluded a consistent, direct association between a pro-inflammatory diet and higher CVD risk and mortality. In subgroup analyses of a meta-analysis, a high DII score was associated with higher risk of myocardial infarction, but not other types of CVD such as stroke and ischemic heart diseases [11]. Furthermore, sex (stronger in women than in men) and region (stronger in Europe, North America, and Japan than Australia) seemed to modify the relationship between DII and CVD. However, it should be noted that the number of studies for these subgroup analyses are low, and thus the results should be interpreted with caution. Studies published after these reviews also reported significant direct association between DII score and CVD risk (estimated from Framingham Risk Score) [172] and CVD mortality [9,173]. Nonetheless, subgroup analysis in our meta-analysis [11] also suggested that the association between DII and CVD risk or mortality is likely independent from the influence of BMI and lifestyle factors such as smoking and physical activity, as the pooled estimates remained statistically significant in studies that adjusted for these factors.

The SUN cohort in Spain is the largest study to date that has examined the relationship between DII and CVD incidence. It was reported that CVD risk was about twice as high (adjusted hazard ratio (HR): 2.03; 95% CI: 1.06, 3.88; total n =18,794) among participants in the highest quartile of DII score, as compared to the lowest quartile [174]. On the other hand, the largest study to date investigating DII vs. CVD related mortality was conducted in a multi-ethnic cohort in the USA (total n = 150,405), which reported an adjusted HR of 1.13 (95% CI: 1.03, 1.23) for men and 1.29 (95% CI: 1.17–1.42) for women, comparing the highest vs. lowest tertiles of DII score [175]. Several potential mechanisms have been proposed to explain the putative influence of pro-inflammatory diet on CVD risk and mortality. For instance, pro-inflammatory diet increases levels of inflammatory cytokines such as TNF-α and IL-1, which may subsequently attract and induce migration of inflammatory cells into vascular tissues [176] or mediate adhesion of white blood cells to the vascular endothelium through increasing expression of cellular adhesion molecules such as cadherins [177]. 

### 5.2. DII and MetS

In the most recent meta-analysis conducted in 2018 [170], only five studies were included for DII vs. MetS relationship, three of which were of cross-sectional design. The overall pooled relative risk (1.01) was not significant (95% CI: 0.82–1.24). However, in studies reporting null associations between DII vs. MetS, some have reported significant associations between DII and components of MetS (e.g., higher DII and lower high density lipoprotein cholesterol in a Polish-Norwegian study [177]), suggesting that different durations of pro-inflammatory exposure were needed for different MetS components [170]. Several studies have been published after the latest meta-analysis, all utilizing a cross-sectional design [41,178,179,180]. The results were again mixed, with three studies reporting higher odds of MetS comparing the highest vs. lowest quantiles of DII scores [8,41,179], while others reported no consistent associations [178,180]. Differences in study settings and participants’ characteristics including age, sex, geographic location, and ethnic background, all potentially leading to differences in the variation of DII, might have explained the disparities in these results. Furthermore, most of these studies were cross-sectional in nature and thus temporality of effect cannot be established, thus limiting causal inference. More definitive results from high-quality prospective studies and trials are needed in this field. 

### 5.3. DII and Obesity

In the sole meta-analysis appraising evidence for DII vs. obesity and BMI, a pro-inflammatory diet is associated with a higher risk of obesity (pooled odds ratio (OR): 1.31; 95% CI: 1.14, 1.50; n = 4 studies) and mean BMI (mean difference: 0.81 kg/m^2^; 95% CI: 0.37, 1.26 kg/m^2^; n = 22 studies) [169]. It is worth noting that there was an extremely high level of heterogeneity for the BMI meta-analysis (*I*^2^ = 94.7%), which was only partially explained by type of dietary assessment (*I*^2^ = 8.4% in three studies using 24-h recalls cf. 98.7% for 19 studies using food frequency questionnaires). Further, three out of four studies concerning obesity included only women, impacting on generalizability to the general population. It also is very challenging to elucidate the direction of causality between inflammation and increased adiposity, with most Mendelian randomization studies suggesting that inflammation is a consequence rather than a cause of obesity [181,182]. Since this meta-analysis, there have been a few more studies looking at this association; one was conducted in Pakistan where significant positive correlations were found between DII score and weight, BMI, waist-to-hip ratio, and body fat percentage [183] and another in Iran where a pro-inflammatory diet was associated with higher BMI z-score, wrist circumference, neck circumference, waist circumference, head circumference and parental BMI [184]. In a longitudinal study from Brazil, a pro-inflammatory diet at baseline was associated with smaller reductions in weight and body fat and poorer dietary quality (reduced consumption of fruits, vegetables, and legumes) six months after bariatric surgery [185]. Complicating this picture is the fact that pro-inflammatory foods tend to be calorically dense, and therefore likely to contribute directly to increased adiposity [15].

### 5.4. DII and T2DM or Gestational Diabetes Mellitus (GDM)

Only one study each has investigated the associations between DII and T2DM in general populations [186] or GDM during pregnancy [187]. High DII scores, indicating pro-inflammatory diets, were significantly associated with a higher risk of T2DM (adjusted OR = 3.02 comparing extreme quintiles) and GDM (adjusted OR = 2.10 comparing extreme tertiles). Another study examining pre-diabetes as the outcome found similar direction of association [188]. Nonetheless, the above-mentioned studies were of cross-sectional/case-control designs and were set in developing countries. High-quality, prospective evidence from other countries with different socioeconomic circumstances is needed. As comorbidities are an important part of population health in which T2DM figures prominently, it is important to note that we found that pro-inflammatory diet, as indicated by higher DII scores, was associated with increased risks of all-cause, CVD, all-cancer, and digestive-tract cancer mortality among pre-diabetic NHANES participants [90]. 

In summary, evidence to date suggests potential benefits of reducing dietary inflammation in lowering CVD risk and its associated mortality, while evidence for MetS, obesity, and diabetes is either conflicting or limited due largely to the paucity of studies, generally small sample sizes, variations in participants’ characteristics such as sex and ethnic background, and the fact that the parameters that define MetS occur earlier in disease development. Further evidence, especially from high-quality prospective studies and carefully designed controlled trials are required before solid recommendations can be issued in the context of MetS, obesity, and diabetes. 

## 6. DII and Respiratory Health 

Growing evidence shows that exposure to an unhealthy diet (in utero and also later in life) might affect respiratory health throughout the life course [189,190]. It has been suggested that pro-inflammatory dietary components might lead to low-grade systemic inflammation, and subsequently, to airway inflammation thus influencing asthma development and severity [191,192]. Anti-inflammatory dietary components might have a protective effect on respiratory outcomes [193,194]. Studies on the associations of the inflammatory potential of the diet, as summarized by the DII score, with lung function and asthma are scarce and have been performed only in a cross-sectional setting. In adults, a small case-control study among subjects showed that 1 unit increase in the DII score was associated with a 70% higher risk of asthma and a 3.44% lower percent predicted Forced Expiratory Volume in 1 s (FEV_1_) [195]. Similarly, a recent large cohort study among more than 30,000 subjects found that only among adults without asthma or wheezing, a higher DII score was associated with a 0.22 and 0.35 lower percent predicted FEV_1_ and Forced Vital Capacity (FVC), respectively [196]. Furthermore, a higher DII score was associated with a higher risk of wheezing, but not with asthma [196]. In children, a pro-inflammatory diet was not associated with current asthma or lung function, but in children with allergic airway inflammation, a higher DII score was associated with a 2.38 fold higher risk of wheezing [196]. 

Although it has been hypothesized that fetal life is a critical period for respiratory health later in life, the programming effect of the maternal DII score in pregnancy on child’s respiratory health has not yet been studied [197]. However, studies on the intake of single pro- or anti-inflammatory nutrients by the mother during pregnancy suggest potential effects of maternal nutrition on child’s respiratory health [189]. The main pro-inflammatory components in the DII score are calorie-contributing nutrients including trans-fat, saturated fat, and cholesterol [27]. Previous studies observed inconclusive results for the association of maternal intake of these nutrients during pregnancy, as well as for the intake of a dietary pattern, which is rich in these fats, with child’s wheezing or asthma [198,199,200,201,202]. Conversely, nutrients mainly derived from fruits and vegetables as well as n-3 fatty acids are the main anti-inflammatory components in the DII score [27]. Although total fruit and vegetable intake in pregnancy was not associated, higher consumption of apples, which are rich in flavonoids, seems associated with a lower risk of childhood wheeze and asthma [202,203]. Furthermore, a randomized controlled trial showed that children of mothers who received supplementation with n-3 long-chain polyunsaturated fatty acids in pregnancy had a risk reduction of 7% for persistent wheeze or asthma [204]. In addition, a higher DII score during pregnancy has been associated, independent of the pre-pregnancy BMI, with higher C-reactive protein levels, which reflect higher maternal systemic inflammation [205]. Higher C-reactive protein levels at birth, but not in early pregnancy, were previously associated with a higher risk of wheezing in pre-school children [206]. Thus, it might be that the inflammatory potential of maternal diet in pregnancy, through systemic inflammation, is a contributing factor to the development of childhood wheezing or asthma. However, the potential underlying mechanisms as well as the long-lasting effects of the inflammatory potential of the diet as summarized by the DII score in different stages of life warrants further investigation. 

## 7. DII and Neurodevelopment 

Child neurodevelopment is caused by a variety of phenomena, influenced by both genetic and environmental factors, as well as interactions between them [207]. The importance of adequate nutrition during fetal life and early childhood with respect to long-term neurodevelopment including cognitive functions, psychomotor abilities, intelligence, and behavior is of increasing interest [208]. It is well established that severe maternal malnutrition during pregnancy and severe deficiency of certain micronutrients can impair child neurodevelopment [209]. On the other hand, some nutrients can be toxic when consumed in excess. However, the impact of more subtle variations in maternal diet quality on child neurodevelopment has been less frequently evaluated. The majority of studies in this field have focused on selected nutritional factors (vitamins, minerals, essential elements, protein, and fatty acids) and not on a diet as a whole. For example, deficiencies in vitamins A and D have been associated with impaired learning and memory, as well as psychomotor and language abilities [209]. Deficiency in group B vitamins has been associated with neural tube defects, delayed language development and impaired motor functioning [209]. Micronutrients including iron, selenium, manganese, and zinc have been shown to have a neuroprotective effect [209,210,211].

Following general recommendations to focus on diet as whole, the meta-analysis by Borge et al. provides a quantitative summary of the existing literature (published between 2004 and 2016) exploring the relationship between maternal diet quality and child cognitive and affective outcomes [208,212,213,214,215,216,217,218,219,220,221,222,223,224,225,226,227,228,229]. The analyzed studies have focused on dietary patterns (most frequently defined as healthy and unhealthy), fish/seafood intake, omega-6/omega-3 fatty acid ratio, saturated fat intake, and dietary fiber, which have been assessed by the use of the food frequency questionnaire (FFQ) and a variety of neurodevelopmental outcomes (externalizing, internalizing, and socio-emotional, cognitive). Based on 18 studies comprising almost 64,000 participants, better maternal diet quality had a small but consistent association with all neurodevelopmental dimensions except for the internalizing dimension, with more reliable results observed for cognitive development. Maternal diet can be a marker for the child’s diet (not systematically controlled for in the majority of the studies), which is a competing exposure that also influences child development. The analysis published by Malin et al. in 2018 also indicates that mothers who consumed more nutritious diets during pregnancy tended to have children with more favorable neurodevelopmental outcomes, while mothers who consumed less nutritious diets and/or higher levels of sodium, saturated fat, and/or sugar during pregnancy had children who tended to perform more poorly on memory, perceptual, and quantitative, verbal and motor tasks [230]. Furthermore, these associations, though similar, were weaker for the impact of childhood nutrition, which, as pointed out by those authors, could suggest that prenatal nutrition plays a greater role in neurodevelopment.

Evidence from human studies has confirmed what was observed in animal models showing that many nutrients are necessary for brain development. The effect of specific nutrients and their deficiency on the following neurodevelopmental processes has been described: neuron proliferation, axon and dendrite growth, synapse formation, pruning and function, myelination, and neuronal apoptosis. Inflammation has been increasingly recognized as an important contributor to central nervous system damage in both the developing child and adult brain. The brain is particularly vulnerable in utero as well as during infancy and early childhood, and insults occurring during these critical periods have the potential to cause long-term damage. Cells within the developing brain use cytokines for autocrine and paracrine signaling, and because many of these cytokines also coordinate the host immune response, normal cytokine-mediated developmental processes are susceptible to disruption by cytokine imbalances [231,232,233,234,235,236,237]. Pro-inflammatory cytokine induction has been shown to adversely affect neurodevelopment whereas anti-inflammatory or regulatory cytokines have been shown to diminish adverse effects. Some neurodevelopmental abnormalities including autism spectrum disorders (ASD) and cognitive impairment have been linked to early life immune activation and inflammation [231]. The DII has been associated with cognitive disorders such as Parkinson’s disease [238,239] as well as normal cognitive decline in aging populations [238,239,240,241,242,243], but there is still a lack of research in relation to child neurodevelopment.

In summary, evidence to date suggests that a better maternal diet quality is associated with a more favorable cognitive development and fewer affective problems in the child.

## 8. DII and Mental Health

The rising prevalence of mental health disorders represents a major public health concern. Worldwide, the prevalence of depression is approximately 350 million [244] and the WHO estimates that more than one in four European adults have experienced a psychological disorder [245]. The Global Burden of Disease Study has highlighted the substantial contribution of adverse mental health to the global burden of NCDs, especially through years lived with disability [244]. Multifactorial processes including, amongst others, biological and environmental factors contribute to an individual’s mental health and well-being [246]. Dietary patterns have been examined in this context. Unhealthy diets characterized by high intake of energy-dense, high-sugar and/or high-fat foods, processed and red meats, alcohol, and refined grains have been associated with increased risk of depression [247,248], whereas healthy diets characterized by a high consumption of fruit, vegetables, whole grains, fish, and lean meats have been linked with reduced depression risk [249,250]. 

Chronic low-grade inflammation also is associated with adverse mental health outcomes [251,252]. Three recent systematic reviews and meta-analyses have examined dietary quality, dietary patterns and dietary inflammation and depressive outcomes [253,254,255]. Based on observational studies adhering to healthy less pro-inflammatory diets appears to be associated with a lower risk of depression. In addition, two recent systematic reviews and a meta-analysis specifically on the DII and depression risk have been published [256,257]. In the systematic review, all but one of the 12 included studies demonstrated an association between dietary inflammation and increased risk of incident depression in adults [256]. The meta-analysis by Wang et al. examined four prospective and two cross-sectional studies (n = 49,584 subjects). They report that individuals with the highest DII scores had a 23% greater risk of depression relative to those with the lowest DII scores. This association was gender specific, being observed in women only [257]. The DII also has been examined in severe mental illness. Using data from the UK Biobank study (n = 68, 879) higher DII scores were observed among those with schizophrenia and major depressive disorder but not bipolar disorder, relative to the controls [258]. 

To date, most of the research examining the relationship between the DII and mental health has focused on depression and depressive symptoms; therefore, this review will focus on those conditions. In female-only studies increased risk of depressive symptoms in adolescents and depression in middle-aged women was observed among those with the most pro-inflammatory diets [259,260]. Gender differences also have been observed in both cross-sectional and longitudinal studies including male and female participants. Examination of prospective associations between the DII and incident depressive symptoms in the SU.VI.MAX (Supplémentation en Vitamines et Minéraux Antioxydants) cohort (n = 3523, mean follow-up 12.6 years) revealed that men, but not women, with higher DII scores had a greater risk of incident depressive symptoms [261]. Further examination of subgroups revealed greater risk among smokers and those who are physically inactive, suggesting that promotion of a more anti-inflammatory diet could confer benefits for these groups. Phillips et al., reported higher risk of depressive symptoms and anxiety (OR 1.70, 95% CI 1.23–2.35 and OR 1.60, 95% CI 1.15–2.24) and lower likelihood of well-being (OR 0.62, 95% CI 0.46–0.83) among adults in the Mitchelstown cohort (n = 2047) with the most pro-inflammatory diet (tertile 3 vs. tertile 1 of E-DII). When stratified by gender these associations were observed among the female participants only [262]. Furthermore, a more pro-inflammatory diet also has been linked with an increased risk of recurrent depressive symptoms in women, but not men, in the Whitehall II study [263]. No gender differences were noted in the examination of the Seguimiento Universidad de Navarra (SUN) cohort study (n = 15,093). After a mean follow-up of 8.5 years depression risk was approximately 1.5 time greater among those in the highest quintile of DII [264], stronger associations were noted among those >55 years and those with cardiometabolic comorbidities. Conversely, examination of data from the NHANES 2007–2012 (n = 11,624) revealed that the association between dietary inflammation and depression was independent of CVD risk as determined by the Framingham risk score [172]. In a cohort of older American adults (n = 3648, mean age 60.6 years) those with the highest DII score (quartile 4) had 24% greater risk of developing depressive symptoms over an eight-year follow-up relative to those with the most anti-inflammatory diet [265]. 

In summary, the evidence to date suggests that a more pro-inflammatory diet is associated with greater risk of depressive symptoms and depression, particularly among women and certain subgroups such as smokers and more physically inactive individuals. Gender effects may be explained by the fact that more studies focus on women and women tend to have higher rates of depression than men [266,267]. Further evidence, especially from randomized controlled trials, of the benefits of a more anti-inflammatory diet in terms of reducing risk of developing depression or improving inflammatory status among individuals with depression is required. 

## 9. DII and Musculoskeletal Health 

Dietary components (including certain micronutrients, vitamins, polyphenols and polyunsaturated fatty acids) and dietary patterns such as the Mediterranean diet have been associated with bone health [268,269,270,271]. Inflammation is also thought to be involved in the development and progression of osteoarthritis, osteoporosis, and fractures [272,273,274,275]. Thus examination of DII in this context has been conducted with a view to improving our understanding of how diet-related inflammation may be related to musculoskeletal health and disease. Thus far, the DII has been examined in relation to risk of osteoporosis, fractures, and falls. As age and female hormonal status are key risk factors for osteoporosis, it is not surprising that much of the research to date has focused on older-aged cohorts and postmenopausal women. 

The Women’s Health Initiative cohort study, the largest US study of postmenopausal women’s health has examined E-DII scores, bone mineral density (BMD), and fracture risk in 161,191 women. Those women with the most anti-inflammatory diets had the smallest BMD loss over 6 years. Stratified analysis revealed greater hip fracture risk but lower total fracture and lower-arm fracture risk among white women aged <63 years with a higher DII score [275]. Using data from the 4th and 5th Korean National Health and Nutrition Examination Surveys (KNHANES, 2009–2011), Na et al. examined associations between DII score and BMD in 2778 postmenopausal women. They report increased risk of total femur osteopenia/osteoporosis among women with the highest DII score (OR 1.27; 95% CI; 1.00–1.62 comparing top vs. bottom DII tertiles) [276]. Results from a small study in postmenopausal Iranian women (n = 160) also suggest that a pro-inflammatory DII score is a risk factor for lower lumbar spine BMD [277]. 

Studies examining both men and women also have been conducted. Examination of associations between DII score with BMD in the US NHANES participants during 2005–2010 (n = 18,318) revealed an inverse association between BMD in different sites with increasing DII scores, which also was related to increased fracture risk [278]. Veronese et al. conducted two separate investigations on DII and bone health [279,280]. The first of these examined 4358 North American adults (mean age 61.2 years) participating in the Osteoarthritis Initiative. Prevalence of experiencing radiographic symptomatic knee osteoarthritis was higher (35.4%) among those with a more pro-inflammatory diet (DII quartile 4) relative to those with a more anti-inflammatory diet (DII quartile 1 (24.0%)). Furthermore, regression analyses, adjusted for potential confounders including age, sex, BMI, physical activity, race, tobacco use, education and income, demonstrated that risk of experiencing radiographic symptomatic knee osteoarthritis was 1.4 times greater among those with the highest DII scores (OR 1.40; 95% CI; 1.14–1.72) [279]. In a case-control study from China, the multivariable-adjusted ORs (95% CIs) for hip fracture across quartiles of DII scores were 1 (reference), 1.42 (1.01, 1.99), 1.63 (1.16, 2.28), and 2.44 (1.73, 3.45) [281].

Gender-specific associations have been observed. Further examination of the Osteoarthritis Initiative participants (3648 adults, mean age 60.6 years) with/at risk of knee osteoarthritis revealed greater fracture risk among women with the highest DII score quintile during eight-year follow-up. No associations were observed in the entire sample or among the men [280]. Cervo et al., examined prospective associations between DII scores and bone health, sarcopenia-related outcomes, falls risk and incident fractures in Australian older adults (n = 1099 aged 50–79 at baseline, with 5 year (n = 768) and 10 year (n = 566) follow-up). They report that among men a more pro-inflammatory diet was associated with lower hip and lumbar spine BMD and greater risk of fractures and falls over 10 years, whereas among women, a more pro-inflammatory diet was associated with greater lower limb muscle quality and reduced fracture risk [282]. 

Not all studies have reported positive associations between DII and bone health. The Brazilian Osteoporosis Study (n = 2269 adults ≥ 40 years old) failed to identify any link between DII scores and low-impact fractures [283]. In further work the relationship between DII scores and muscle mass and strength were examined in Chinese children (n = 466, age six–nine years). The authors reported negative associations between DII and total body skeletal muscle mass, appendicular skeletal and lean mass, and no association with grip strength [284]. Potential mechanisms linking dietary inflammation with bone health have been suggested including immune dysregulation characterized by chronic inflammation, which may promote overactivation of osteoclasts, reduction in osteoblast activity, changes in the extra-cellular matrix driving cartilage loss, and joint degeneration [285,286,287,288]. 

In summary, to our knowledge no meta-analyses have been conducted on the DII and bone/musculoskeletal health to date. Examinations of the findings from individual observational and longitudinal cohort studies suggest an unfavorable influence of a pro-inflammatory diet on fracture risk, BMD and osteoporosis. However, some limitations can be identified including a focus on postmenopausal women and ageing cohorts, thereby limiting generalizability of the findings. Where both sexes have been examined the noted sex differences suggest that higher DII scores may be more detrimental to musculoskeletal health in older men. Our understanding of the mechanisms underlying the associations between the early environment and future bone health is evolving. It is possible that nutrients and dietary inflammation may have a direct effect on bone mineralization, for example, maternal pregnancy 25-hydroxyvitamin D concentration is associated with umbilical cord venous serum calcium concentration; expression of a calcium transporter in the placenta is positively associated with neonatal bone mass [289]. However, there is increasing evidence for indirect mechanisms, involving epigenetic signaling, for example perinatal methylation at the retinoid X receptor alpha locus (a key part of vitamin D signalling) is reduced by maternal gestational vitamin D supplementation, and is associated with offspring bone mass [290,291]. Future work examining the relationship between early life or childhood dietary inflammation on future bone health could provide important insights. 

## 10. DII and Intergenerational Health 

Pregnancy normally induces an anti-inflammatory response profile, which is tightly regulated from conception to delivery [292]. There is, however, little evidence that such an anti-inflammatory immune switch occurring during pregnancy may attenuate obesity-driven inflammation. Rather, it has been suggested that dysregulated innate immune profiles could be involved in fetal programming caused by maternal obesity [293]. Indeed, obesity-induced maternal inflammation or meta-inflammation may have a direct effect on fetal development, provided that maternal-derived inflammatory components such as cytokines, activated immune cells, or maternal metabolites are transferred across the placenta into fetal circulation. Such maternal inflammation also could have indirect effects on the fetus by modulating placental capacity to transfer nutrients to the fetus [294]. Increased cytokine levels have been detected in the placenta and cord blood of babies born to obese mothers [295]. Thus, maternal meta-inflammation might cause altered metabolic health in the offspring either by direct transfer of cytokines to the fetus or by altering the placental nutrient flow [296] or via epigenetic mechanisms [297]. 

The Barker hypothesis proposes that maternal in utero and early-life nutrition may permanently impact offspring physiology and alter their health trajectory [298]. This impact may be intergenerational and may be mediated by epigenetic mechanisms. Maternal *intra-uterine* environment, through epigenetic modulation, has shown to be an important determinant of offspring metabolism and health outcomes [299]. In a recent Gambian study, women conceiving in the rainy season had higher plasma concentrations of folate, vitamin B-2, betaine, cysteine and lower concentrations of dimethylglycine, pyridoxal-5′-phosphate, and homocysteine. Furthermore, there were associations between maternal 1-carbon biomarkers and offspring DNA methylation at metastable epialleles [300,301]. These findings led the authors to suggest that if underlying nutritional status acts as an effect modifier for DNA methylation, then this would be applicable to populations with differing dietary patterns. In this context, maternal dietary inflammatory potential could also have its place within the potential physiological mechanisms explaining early nutritional programming of offspring birth outcomes and later health and disease. 

Thus far, limited investigation of maternal DII or its potential influence on offspring epigenetics, birth outcomes or childhood health has been conducted and results are conflicting. Two separate investigations of Project Viva, a cohort consisting of mother-child pairs, revealed that a more pro-inflammatory diet during pregnancy was associated with maternal systemic inflammation, lower birth weight for gestational age, and infant adiposity [204,302]. The Healthy Start Study (n = 1078 mother-neonate pairs) reported that higher DII scores in obese, but not lean or overweight, mothers were associated with increased neonatal adiposity [303]. The Newborn Epigenetic Study (NEST) cohort (n = 1057 mother-child pairs) examined associations between maternal E-DII during pregnancy and offspring birth outcomes. Further analysis of subsets included methylation of differentially methylated regions of imprinted genes involved in fetal growth and development (n = 338) and maternal cytokines (n = 105). While no associations between maternal E-DII and offspring birth weight, methylation, or maternal cytokines were found, higher E-DII scores were associated with greater risk of pre-term birth of female offspring and with cesarean delivery for women with a BMI in the obese range [304]. Ethnic diversity, use of either the DII and E-DII, and maternal weight status during pregnancy may contribute to the disparity between studies. 

In summary, a large body of evidence, particularly from animal studies, shows that food components have the ability to modulate epigenetic markers both directly and indirectly through the in utero environment from mother to offspring [299]. While selected micronutrients or food components have been examined in human studies, there is a lack of research on dietary patterns and specifically the DII. Thus, further investigation regarding the potential influence of early life exposure to a pro-inflammatory environment on long-term and intergenerational health and disease is necessary. 

## 11. Conclusions and Future Directions

In this review, we examined and synthesized findings from a very large and rapidly growing body of research investigating associations between dietary inflammatory potential, determined by the DII, and NCD risk. The evidence suggests that a more pro-inflammatory diet in adults is associated with increased risk of certain cancers, CVD and its associated mortality, adverse mental health, and musculoskeletal disorders. Overall, results support a role for dietary inflammation in the pathophysiology of these conditions. These findings highlight the potential benefits of transitioning to a more anti-inflammatory/less pro-inflammatory diet to decrease disease risk. The evidence regarding DII and respiratory health, neurodevelopmental outcomes, MetS, obesity and diabetes is either conflicting or limited. Thus future research investigating the potential influence of dietary inflammatory status on these conditions is warranted. Limitations of the evidence are that it is based mostly on observational studies so causality cannot be firmly established because the temporality criterion of the Criteria for Judging Causality is often not satisfied [305,306]. Future studies, including adaptive/pragmatic and randomized controlled trials [307,308], are required to provide additional evidence. The evidence of positive associations across a wide array of studies examining different outcomes in diverse populations indicates that the computation of the DII/ E-DII is robust with respect to dietary assessment, especially different FFQs.

The recent development and validation of the children’s DII (C-DII) presents new opportunities to investigate similar diet-health relationships in childhood. Such studies may provide important insights, particularly from developmental origins of health and disease perspective. Given that early-life nutritional exposure may exert effects on long term health and that these effects may also be intergenerational, we eagerly await the findings of the ALPHABET study (“Early life programming of childhood health: a nutritional and epigenetic investigation of adiposity and bone, cardiometabolic, neurodevelopmental and respiratory health”), which is investigating early-life nutritional programming of childhood health. The ALPHABET consortium consisting of several European birth cohorts (>65,000 participants) [309,310,311,312,313,314,315] is specifically examining maternal DII during pregnancy and associations with offspring birth outcomes, childhood health outcomes including adiposity and bone, cardiometabolic, neurodevelopmental and respiratory health, and epigenetics. Improving our understanding of nutritional programming of childhood health may help inform development of more effective evidence-based public health strategies for example, with an emphasis on advocating a healthy anti-inflammatory diet in pre-pregnancy, pregnancy and early postnatal life, to improve both mother and offspring health and attenuate development of adverse health outcomes over the life course and potentially in future generations. It is important to note that diet is only part of the equation and other lifestyle factors especially sedentary behavior and physical activity play key roles in modulating inflammation and health. Thus more holistic assessment of healthy lifestyle behaviors, including dietary inflammation, may inform the development of more effective healthy lifestyle interventions, which may open up new avenues of investigation.
